# Slow moving neural source in the epileptic hippocampus can mimic progression of human seizures

**DOI:** 10.1038/s41598-018-19925-7

**Published:** 2018-01-24

**Authors:** Chia-Chu Chiang, Xile Wei, Arvind Keshav Ananthakrishnan, Rajat S. Shivacharan, Luis E. Gonzalez-Reyes, Mingming Zhang, Dominique M. Durand

**Affiliations:** 1 Department of Biomedical Engineering, Case Western Reserve University, Cleveland, Ohio 44106 USA; 2 Present Address: School of Electrical and Information Engineering, Tianjin University, Tianjin, 300072 China

## Abstract

Fast and slow neural waves have been observed to propagate in the human brain during seizures. Yet the nature of these waves is difficult to study in a surgical setting. Here, we report an observation of two different traveling waves propagating in the *in-vitro* epileptic hippocampus at speeds similar to those in the human brain. A fast traveling spike and a slow moving wave were recorded simultaneously with a genetically encoded voltage sensitive fluorescent protein (VSFP Butterfly 1.2) and a high speed camera. The results of this study indicate that the fast traveling spike is NMDA-sensitive but the slow moving wave is not. Image analysis and model simulation demonstrate that the slow moving wave is moving slowly, generating the fast traveling spike and is, therefore, a moving source of the epileptiform activity. This slow moving wave is associated with a propagating neural calcium wave detected with calcium dye (OGB-1) but is independent of NMDA receptors, not related to ATP release, and much faster than those previously recorded potassium waves. Computer modeling suggests that the slow moving wave can propagate by the ephaptic effect like epileptiform activity. These findings provide an alternative explanation for slow propagation seizure wavefronts associated with fast propagating spikes.

## Introduction

Seizures are known to propagate and the localization of the focus is essential to a successful therapeutic intervention. Yet finding the source of the seizures is a difficult and often unsolved problem. Seizure foci are difficult to observe as they can move or can arise from micro seizures in multiple locations^1,2^. In patients with epilepsy, identifying multiple foci in temporal lobe is an additional challenge and the mechanism of seizure generation remains unknown^3^. It has been shown recently in the human brain that there are two types of traveling waves, a slow and a fast wave (about 10 times faster) associated with propagating seizure activity^4^. Yet the origin of these waves and their mechanism of propagation are difficult to be determined *in-vivo*. Similar waves at similar speeds have been observed *in-vitro* in the hippocampus under epileptic conditions^5,6^ and the cortex under normal physiological conditions^7^. We have observed fast spontaneous 4-AP-induced inter-ictal spikes propagating at a speed of approximately 0.1 m/s as well as the source of the spike propagating simultaneously at a slower speed of approximately 0.01 m/s. The mechanism of propagation for the fast traveling spike in the 4-AP induced ictal activity has already been characterized as non-synaptic and consistent with ephaptic propagation^5,8^. The slow moving neural source could not be detected directly by electrical recording. However, the path of the source could be inferred from isochrone maps obtained from the generated fast traveling spikes. The speed of the source was estimated by using Doppler calculations^6^.

In this study, the neural circuit spatiotemporal dynamics of this source were imaged using a genetically encoded voltage-sensitive fluorescent protein (VSFP Butterfly 1.2) to directly trace the slow moving wave induced by 4-AP in the non-epileptic hippocampus. The VSFP Butterfly 1.2 shows a reliable response to the membrane potential, fast kinetics, and high signal-to-noise ratio^9^ and diminished toxic effect compared to voltage sensitive fluorescent dyes^10^. Thus voltage-sensitive fluorescent proteins are suitable to track the propagation of neural activity along the longitudinal plane of the hippocampal slice.

In addition, it has been shown that 4-AP can induce a slow neuronal calcium oscillation^11^. Therefore, we also tested the hypothesis that a neural calcium wave is associated with the focal region and propagates with the slow wave. We tested this hypothesis by means of a calcium-sensitive fluorescent dye, Oregon Green 488 Bapta-1 (OGB-1). OGB-1 and other calcium-sensitive dyes, such as fura-2, have been used in previous studies to detect neural activity in the neural tissue^12^. OGB-1 can detect changes in calcium concentration in small dendritic compartments^13–15^ and therefore has been proposed as a tool for tracking activity that is not visible using traditional electrophysiology methods^16^. Finally, we developed a computational neuronal network model to simulate the two traveling waves simultaneously and compare their properties to test the possible mechanisms of generation and propagation of these waves.

## Results

### 4-AP induced fast traveling spike can be imaged propagating through the hippocampus

**Figure 1:**
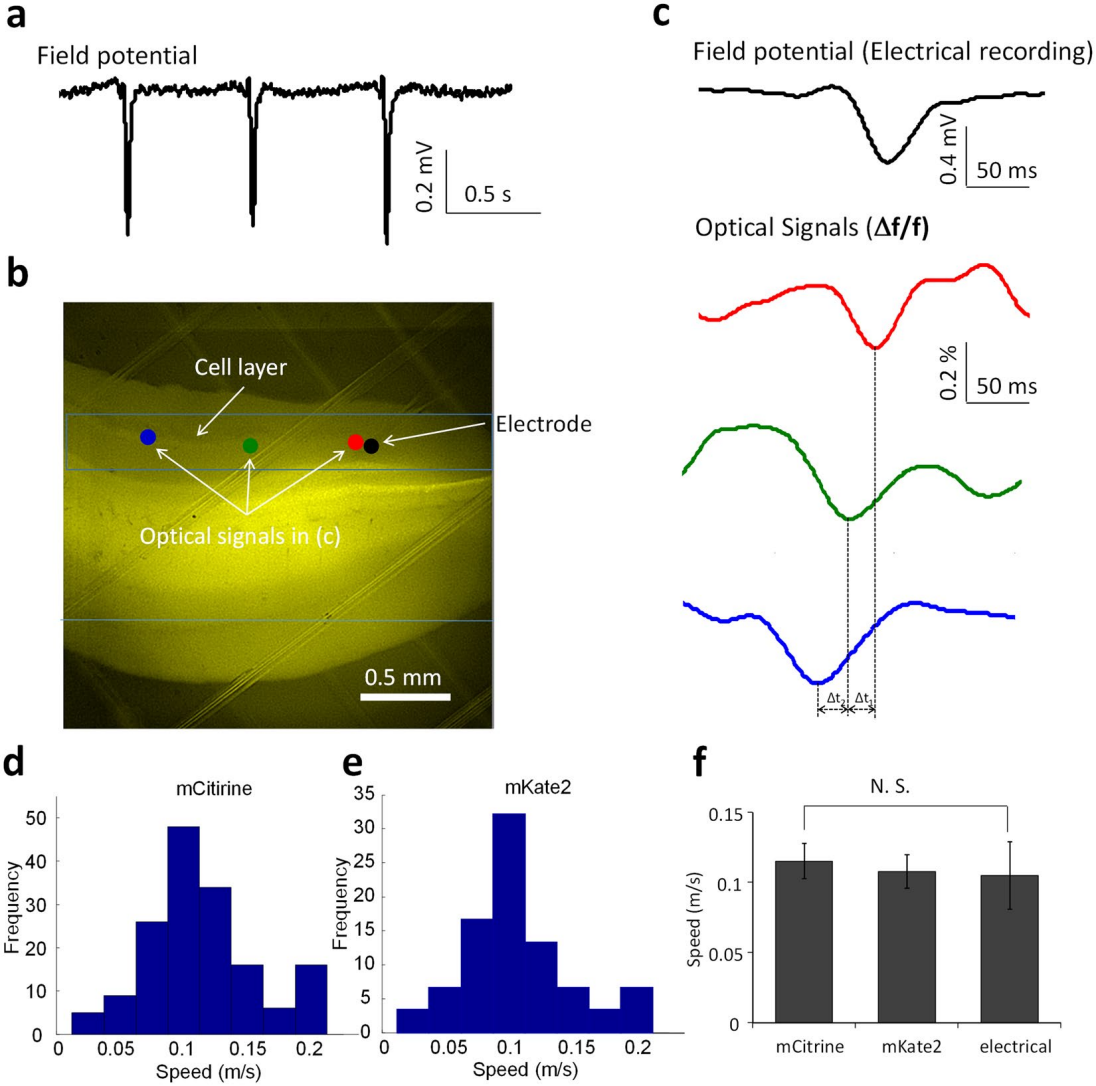
Optical recording characteristics of fast traveling spikes in VSFP butterfly 1.2 transgenic mice. (**a**) Epileptiform activity from local field potentials recording in the longitudinal hippocampal slice with the administration of 4-AP. (**b**) Fluorescent image of the VSFP butterfly 1.2 transgenic mice. (**c**) Electrical recording (local field potential) of the fast traveling spike from the black spot in (**b**) and optical signals (fluorescent intensity) change over time from 3 different regions of interest, red, green, and, blue spots in (**b**). (**d**) Histogram of propagating speed of the fast traveling spikes calculated from mCitrine optical signals. (**e**) Histogram of propagating speed of the f fast traveling spikes calculated from mKate2 optical signals. (**f**) Average speeds from mCitrine, mKate2 optical recording and the electrical recording. The speed is ~0.1 m/s and t-test shows that there is no significant difference between two different optical signals. N.S.: no significance.

4-AP induced spikes can propagate rapidly both in the transverse and longitudinal directions through the unfolded hippocampus but their path is not clearly known because of the poor spatial resolution of the electrode array^5^. To track these spikes with the improved spatial resolution we used voltage indicators with longitudinal slices from transgenic mice carrying voltage-sensitive fluorescent protein (VSFP Butterfly 1.2) immersed in the 100 μM 4-AP/aCSF to induce the inter-ictal spikes. In addition to recording the local field potentials (Fig. 1a), a high speed camera (C11440, Hamamatsu) was used to acquire image sequences to reconstruct the spatiotemporal activity (Fig. 1b). The VSFP Butterfly 1.2 exhibited a voltage-dependent decrease of Δf/f in mCitrine (donor) and an increase in mKate2 (acceptor) emission corresponding to membrane depolarization^9^. Following the calculation of the intensity change of fluorescence (Δf/f) in the region of interest (ROI), the optical signals in different regions of the hippocampal slice were reconstructed as temporal signals. The optical signal from mCitrine was similar to the local field potential indicating that the fast spikes follow the cell layer (Fig. 1c). To calculate the speed of these propagating events, the optical signals at 11 different positions along the cell layer were processed to determine the delay with cross correlation calculation for each single spike events. The speed of fast traveling spikes estimated by optical signals in two different regions of interest from mCitrine was in the range between 0.025 and 0.2 m/s and was similar to the speed obtained from mKate2 imaging (Fig. 1d,e). In summary, the average speeds of the fast traveling spikes induced by 4-AP/aCSF solution estimated from mCitrine and mKate2 were 0.12 ± 0.01 m/s (n = 170 events) and 0.11 ± 0.01 m/s (n = 130 events), respectively. The speed of the fast traveling spikes was also estimated by the electrical signals that revealed a speed of 0.11 ± 0.02 m/s (n = 100 events). A one-way ANOVA followed by a *post hoc* test showed that there was no significant difference between speeds estimated from both optically and electrically recorded signals. There was also no significant difference between the two measurements to calculate the speed from the optical signals (Fig. 1f). The speed of the fast traveling spike induced by 4-AP in the longitudinal slice was similar to the speed of inter-ictal spike induced by 4-AP in the unfolded hippocampus from our previous study (0.1 m/s)^5^.

**Figure 2:**
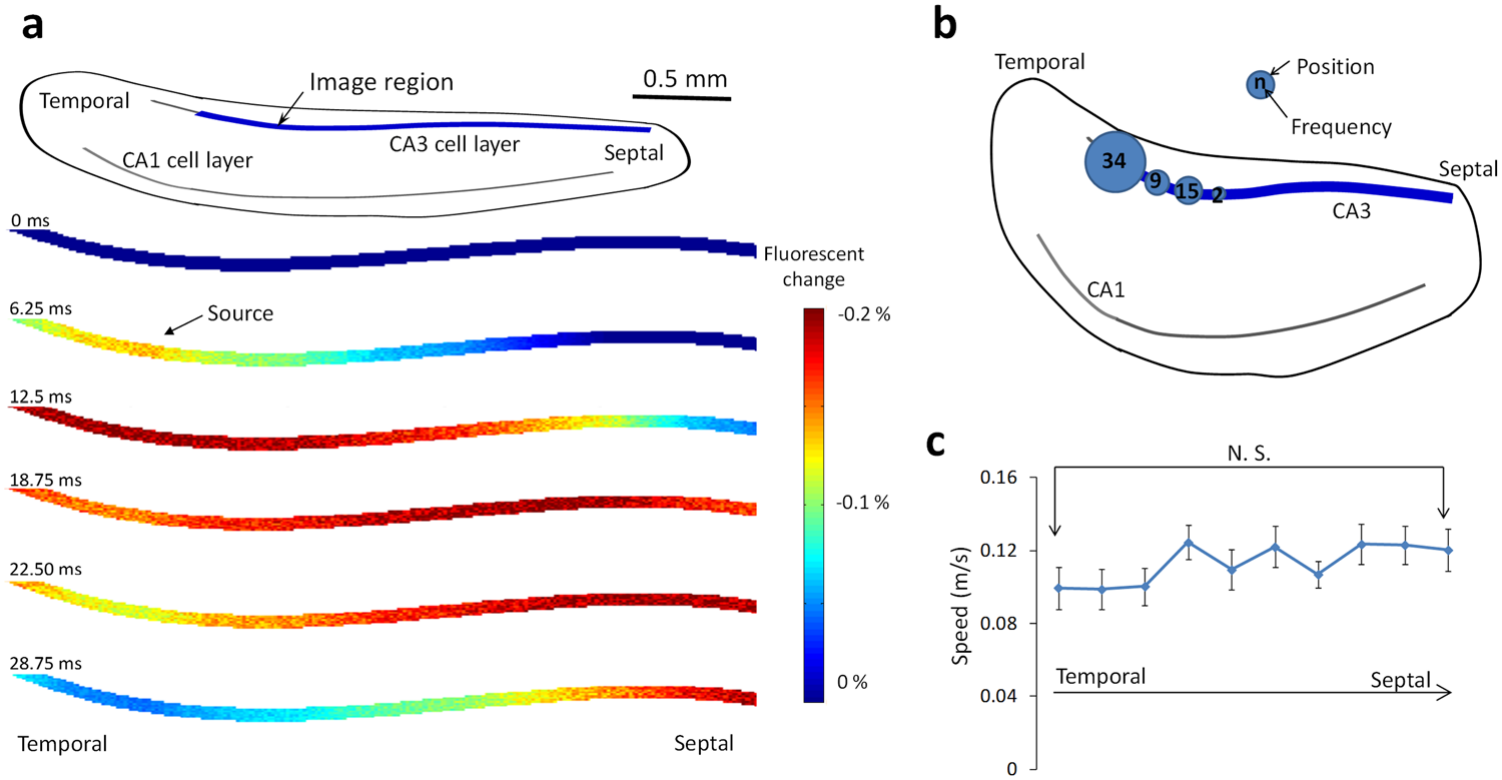
Spatio-temporal characteristics of the fast traveling spikes. (**a**) Example of a fast traveling spike. A spike initiates at a temporal segment of cell layer and propagates with two different directions to the other parts of the hippocampus. (**b**) Positions of the source. The majority of spikes originate in the temporal segment of the hippocampus. (**c**) The speed of the fast traveling spike along the septotemporal axis was not significantly different by one-way ANOVA test (n = 60 events).

To study the spatiotemporal characteristics of the fast traveling spikes, image sequences recorded during each single spike event were analyzed. We observed several consistent features. First, the fast traveling spikes propagated through the cell layer. In some cases, the spike originated within the imaging window and the fast traveling spikes could be observed to propagate in two different directions (Fig. 2a). Second, the origin of the wave could be traced in each image sequence and the result can be seen in Fig. 2b. Over 90 percent of spikes initiated in the temporal area of the hippocampal slices (n = 60 events). Finally, when the speed of the fast traveling spikes was traced along the cell layer, one-way ANOVA test shows there was no significant difference between the speeds measured in the temporal and septal segments of the hippocampus (Fig. 2c).

### 4-AP also induces a slow moving wave propagating along septotemporal hippocampus

**Figure 3:**
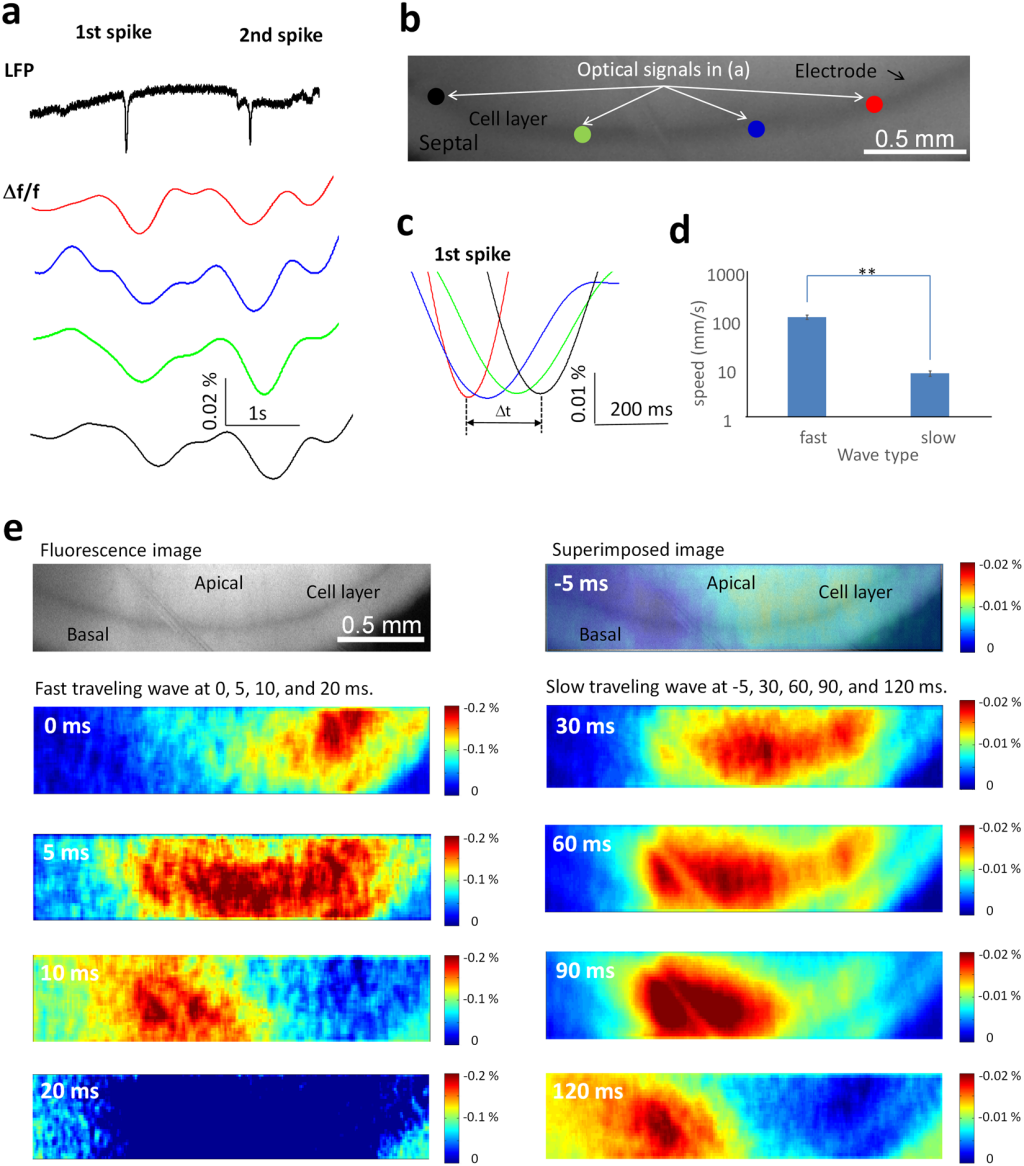
Optical recording characteristics of slow moving wave in butterfly 1.2 transgenic mice. (**a**) Local field potential of the fast spike and fluorescence intensity changes over time of the slow moving wave from 4 different regions of interest, red, blue, green, and, black spots of the fluorescence image (**b**) in one hippocampal slice. The slow moving wave is accompanied by the spike from local field potential recording. (**c**) The expanded window of optical signals in (**a**) shows that the slow wave travels in 4 different regions of interest along the cell layer. (**d**) The average speeds of the fast and slow moving wave. **p < 0.01. (**e**) The propagation map of the fast traveling spike in the left column and the slow moving wave in the right column. The top left figure is the whole-field fluorescence image. The actual length of the image is 2.6 mm.

The existence of a neural source slowly propagating in the hippocampus was previously inferred from a microelectrode array data since it could not be observed electrically^6^. To observe directly this slow moving wave along the cell layer, optical signals from different locations were reconstructed in the time domain and filtered with a 1 Hz low-pass filter (Fig. 3a). The filtered signals reveal that a slow drifting wave was always present when a fast traveling spike was recorded from local field potentials (Fig. 3a). When measuring the time delay of the optical signals from different positions, it was found that this drifting wave traveled through the cell layer at a slower speed compared to the fast spikes (Fig. 3b,c). When the fast traveling spike was detected by electrical recording, a slow moving wave appeared and propagated from the temporal region to the septal region of the hippocampus (Fig. 3a and e). The propagating speed was 0.0077 ± 0.0005 m/s (n = 60 events, Fig. 3d) and this speed is significantly different from the speed of the fast traveling spikes (paired-t test, p < 0.05). Therefore, these experiments confirm the hypothesis that propagating activity in the longitudinal plane comprises of two distinct components: a fast spike traveling along the cell layer and a slow moving wave propagating simultaneously in the same region.

For each spike event in the field potential, optical signal revealed both a fast traveling spikes and a slow moving wave. To understand the relationship between the fast and slow moving waves, the optical signals from the same electrical spike event are plotted simultaneously in Fig. 3e. The figure shows a slow moving wave initiated at −5 ms and triggering a fast traveling spike propagating through the tissue shown at 0, 5, 10 and 20 ms. Simultaneously, the slow wave continues to propagate slowly as shown at 30, 60, 90 and 120 ms. This observation that the slow wave triggers the fast wave was further tested by a hippocampal computational model. Simulated intracellular potentials show the slow wave can trigger the fast propagating wave. These data will be described in detail in the later paragraph. These experimental and simulated data indicate that the fast traveling spikes were triggered by the slow moving wave as previously observed in the intact hippocampus preparation^6^.

### Slow moving wave is a neuronal calcium wave

**Figure 4:**
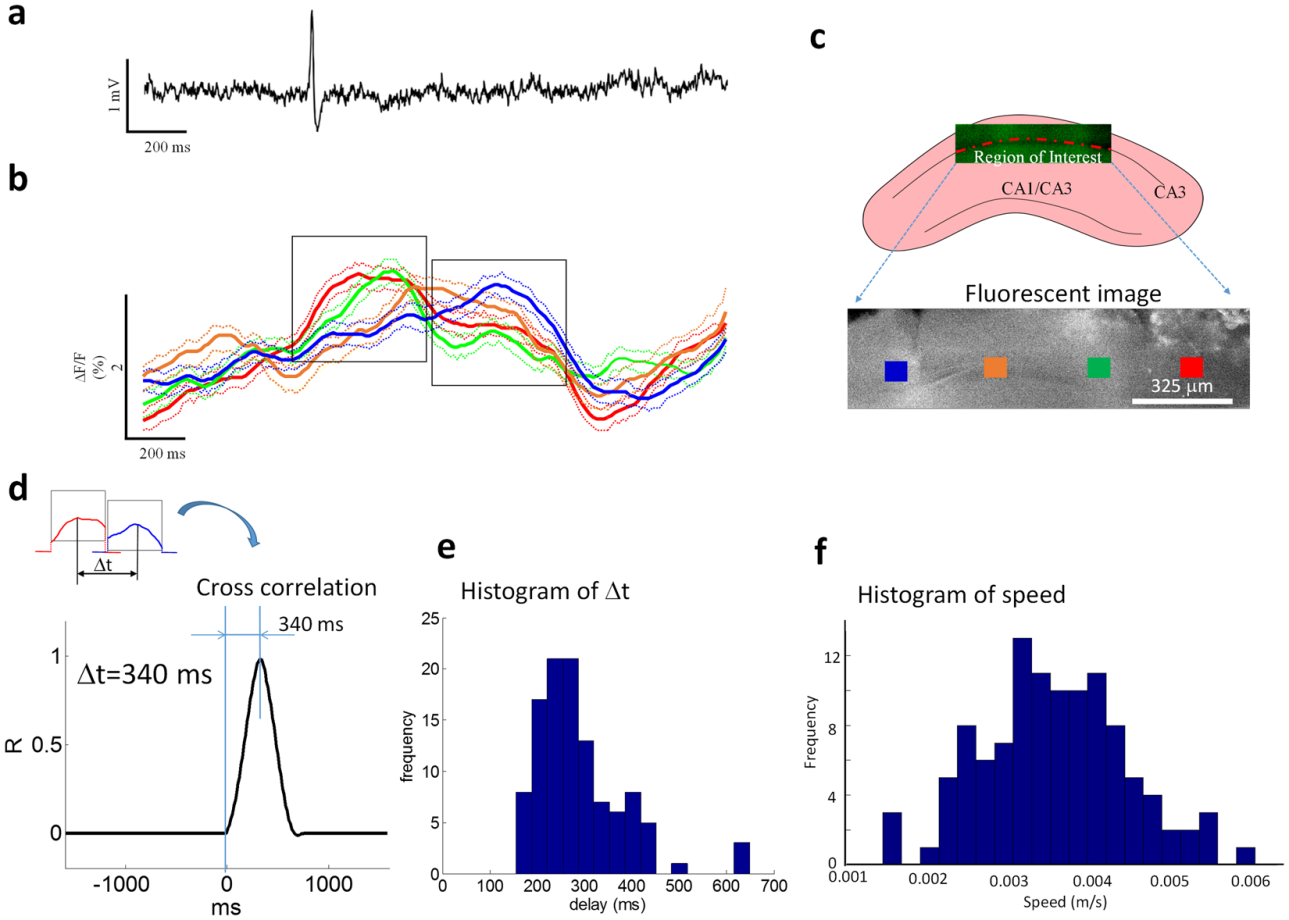
Slow moving waves recorded using calcium imaging. (**a**) Local field potential recording from the slice in the calcium imaging experiments. (**b**) Optical signals of the slow moving wave from 4 different regions of interest in (**c**) of the hippocampal slice. Solid line: mean value. Dash line: mean +/− SD. (**c**) An example of imaging region in a longitudinal hippocampal slice and selected regions of interest for further analysis in the fluorescent image. (**d**) An example of the delay estimated by cross-correlation calculation between two optical signals (red and blue traces). (**e**) The histogram of estimated delay based on cross-correlation calculation from 122 events of slow moving waves. (**f**) The histogram of the speed of the slow moving wave detected by calcium imaging.

To characterize the nature of the slow moving wave, several hypotheses were tested in the following experiments. First, we hypothesized that the slow moving wave is associated with an increase in intracellular calcium concentration within the slow moving wave since 4-AP can induce a calcium oscillation^11^. To observe the calcium dynamics of the slow moving wave directly, longitudinal slices were incubated in a solution containing OGB-1 calcium dye and placed in an imaging chamber with 4-AP/aCSF at a flow rate of 0.05 ml/s. Neurons were stained using the AM ester of OGB-1. Figure 4c shows a typical calcium imaging window in a longitudinal hippocampal slice. The imaging window location was focused on the center of the slice with the cell layer in the field of view. To monitor the fast traveling spikes, an extracellular recording electrode was placed close to the cell layer, approximately 20 µm below the surface and Fig. 4a show an example of fast traveling spike by electrical recording. To obtain the slow moving wave, optical signals in four different regions of interest from calcium imaging were calculated as fluorescent intensity change (∆F/F) in the time domain (blue, orange, green, and red squares in Fig. 4c). Each optical signal from different regions of interest is made up of the averaged intensity of an array of 8 × 8 pixels (mean ± SD) is plotted versus time (Fig. 4b). The optical signals analyzed were in the range of 0.5–2% (Fig. 4b). The time-to-peak of these changes were in the range of 0.05 ± 0.02 s, and the subsequent fall time was in the range of 0.55 ± 0.4 s (n = 112 events sampled from 14 slices with equal number of events). The statistical analysis shows that the peak of optical signal intensity generated by the slow moving wave is significantly higher than the baseline (1.3 ± 0.4% compared to baseline RMS level of 0.3 ± 0.2% with SNR of 4.3 fold (paired-t test, p < 0.05, n = 112 events from 14 slices with equal number of events). The simultaneous recording of the optical slow moving wave and electrical fast traveling spike were also analyzed by Chi-squared test. When hippocampal slices were immersed in 4-AP/aCSF solution, most of optical slow moving waves would accompany an electrical fast traveling spike (p < 0.01).

To study the propagation of the slow moving wave, optical signals from two different regions of interest were compared to calculate the delay with cross-correlation calculation^5,6^. Figure 4d shows an example of cross-correlation calculation from two optical signals in two different regions of interest (blue and red squares in Fig. 4c). The cross-correlation function revealed a time delay of 340 ms (R-value of 0.95). Figure 4e shows a histogram of delay calculated by cross-correlation calculation from 112 different events and the statistical analysis shows that the delay is significant when signals are compared from two different regions of interest (paired-t test, p < 0.05). The speed of propagation was in the range of 0.001 and 0.007 m/s and the mean was 0.004 ± 0.0009 m/s (Fig. 4f). The direction of propagation of the source associated with the spike was always from the temporal to the septal region of the hippocampus (112 of 112 events).

### NMDA-antagonists block the fast traveling spike but not the slow moving wave

**Figure 5:**
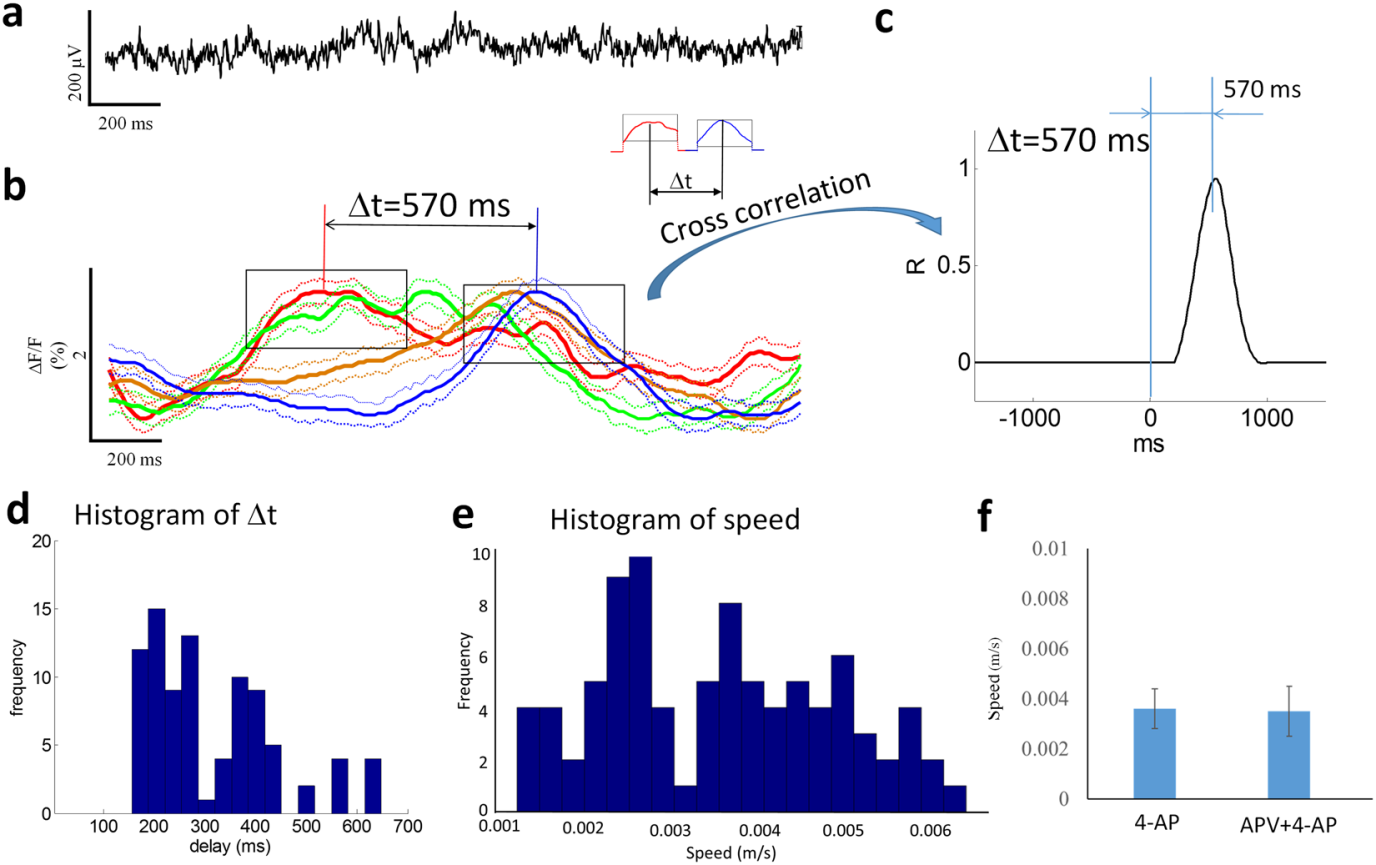
The slow moving wave is independent of NMDA receptors. (**a**) Local field potential recording from the slice with the administration of APV + 4-AP/aCSF shows fast traveling spike is eliminated. (**b**) Optical signals of the slow moving wave from 4 different regions of interest in the hippocampal slice. The slow wave can still propagate in the presence of NMDA receptor blocker, APV. Solid line: mean value. Dash line: mean + /−SD. (**c**) The cross-correlation calculation reveals that there is a delay of 570 ms between two optical signals (red and blue trace in c). (**d**) The histogram of estimated delay based on cross-correlation calculation from 88 events of slow moving waves. (**e**) The histogram of the speed of the slow moving wave in the presence of APV. (**f**) The speed does not change before and after the application of APV by Mann–Whitney U test.

The fast traveling spike was observed to propagate in zero-calcium solutions in a previous study but still required the activation of postsynaptic NMDA (but not AMPA) receptors with bath glutamate^7^. We then tested the NMDA sensitivity of the slow moving wave. We hypothesized that the NMDA receptor can only underlie the fast traveling spike but not the slow moving wave based on their relative speed. This hypothesis predicts that the slow moving wave should still propagate in the absence of spikes and in the presence of an NMDA receptor blocker (APV). This hypothesis was tested by placing the slice in the imaging chamber with perfusion of a solution of 50 μM APV + 4-AP/aCSF. Examples of electrical and optical recordings in the condition of 50 μM APV + 4-AP/aCSF are shown in Fig. 5a,b. In all 4 slices with the presence of the NMDA blocker, the fast traveling spikes were blocked in the electrical recordings (Fig. 5a). However, slow moving waves were still detected when the optical signals from four different regions of interest (Fig. 5b). The slow moving wave has a rise time to peak of 0.05 ± 0.01 s and the decay time of 0.53 ± 0.4 s (n = 88 events from 4 slices with equal number of events). The four optical signals from four different regions of interest revealed that the slow moving wave could still propagate with the presence of NMDA antagonist, APV (Fig. 5b). To study the propagation, two optical signals were compared by calculating cross-correlation to obtain the delay between two signals (Fig. 5c). Figure 5d shows a histogram of delay from cross-correlation calculation and the statistical analysis shows that the delay is significant between two signals from two different regions of interest (Mann–Whitney U test, p < 0.05, n = 88 events from 4 slices with equal number of events). Figure 5e provides the histogram distribution of the speeds of propagation under APV + 4-AP/aCSF. The speed of propagation was 0.004 ± 0.001 m/s (n = 88 events) and t-test show that there was no significant difference between solutions with and without the NMDA receptor blocker (Fig. 5f). Therefore, these experiments confirm that the slow moving wave is independent of NMDA receptor.

### Slow moving wave is independent of ATP release

**Figure 6:**
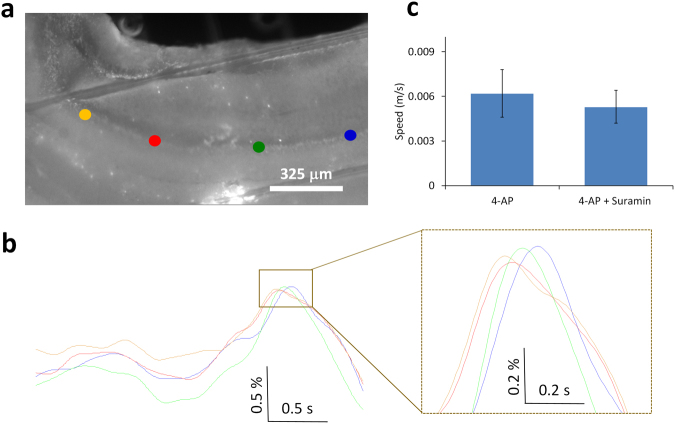
The slow moving wave is independent of ATP release. (**a**) Fluorescence image from the hippocampal slice with the administration of suramin + 4-AP/aCSF. (**b**) Optical signals from 4 different regions of interest, blue, green, red, and yellow spots in (**a**), show the wave can still travel without changing the speed in (**c**).

Propagating astrocyte calcium waves have been shown to involve the release of ATP activating neighboring purinergic receptors, causing an increase of intracellular calcium, and generating a calcium wave^17^. Therefore, we next tested the hypothesis that the slow moving wave is mediated by ATP release. Three hippocampal slices were perfused with 4-AP/aCSF first to generate inter-ictal activity and then 100 μM suramin plus 4-AP/aCSF was added to block the purinergic receptors of the P2Y group^18,19^. The spike could still be detected in the local field potentials. By using the OBG- 1 calcium dye, the slow moving wave could also be observed in the optical signals (Fig. 6a). The slow moving wave continued to propagate in the presence of suramin and a paired t-test shows that the speeds before and after the application of suramin were similar (Fig. 6b,c). These experiments show that the slow moving wave is not mediated by ATP release.

### Fast traveling spike and slow moving waves can be explained by a computational neuronal network model

**Figure 7:**
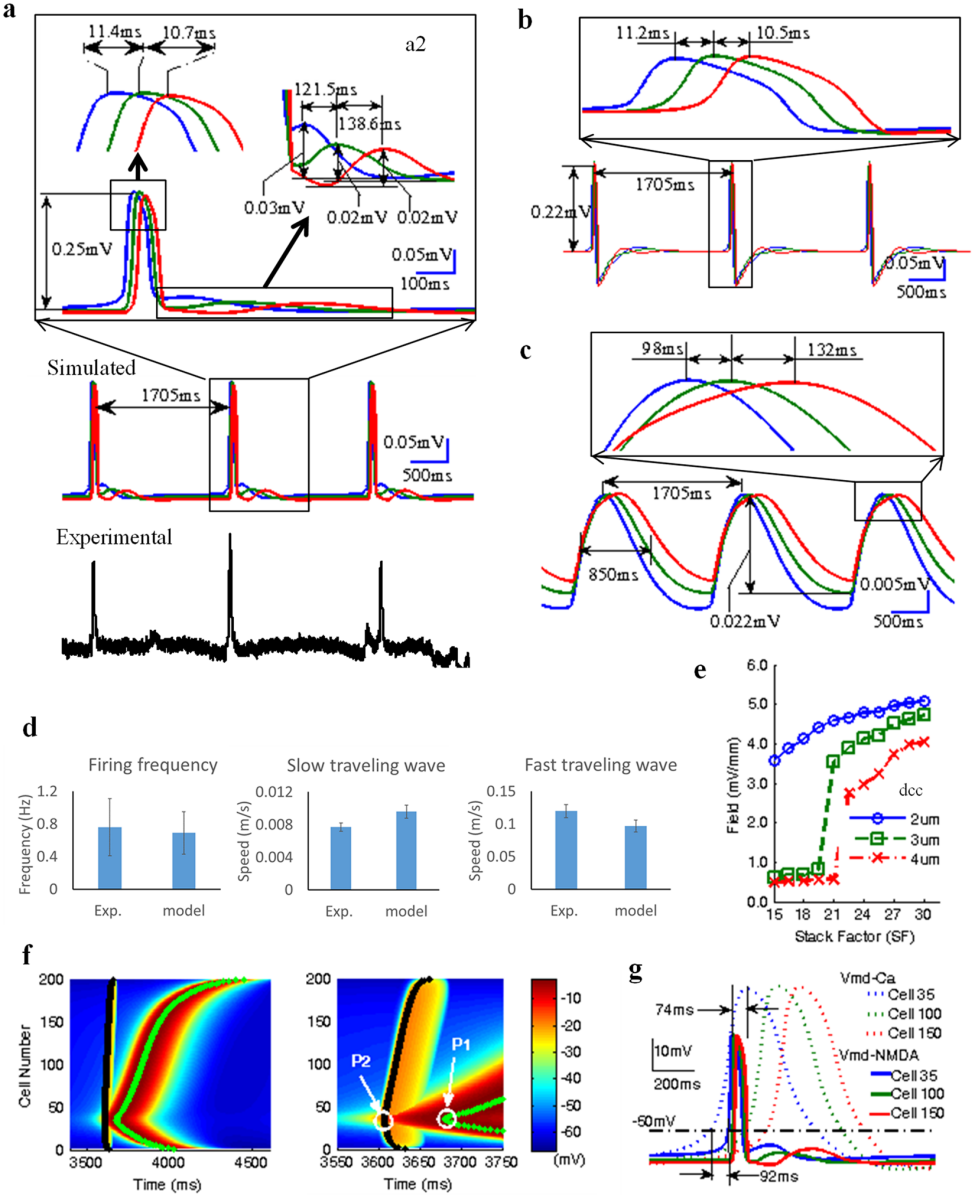
Co-existence of calcium-dependent slow and NMDA-dependent fast propagations in the simulated hippocampal network. (**a**) Experimental and simulated local field potentials. The blue, green and red lines represent the oscillations recorded from three top virtual electrodes respectively. The local zoom of a simulated spike event can show the coexisting fast and slow oscillation propagations. (**b**,**c**) Filtered local field potentials can reveal the fast traveling spike by using 1 Hz high-pass (**b**) and the slow moving wave by a 1 Hz low-pass Butterworth filter (**c**). (**d**) Comparison of the, firing frequency and speeds of fast and slow moving waves, between experimental data and simulated data. (**e**) The curves of the simulated extracellular field amplitudes v.s. stack factor (SF) in three different cell-to-cell distances (dcc): dcc = 2, 3, 4 μm. (**f**) Simulated intracellular membrane potentials in the apical and basal dendritic compartments of cells in the network. The green line and black line represent the peak trajectories of calcium-dependent spikes and NMDA-dependent spikes among all cells from cell#1 to cell#200 along longitudinal direction respectively. In the time expanded window, P1 represents the first peak of the calcium-dependent spike during slow wave propagation and P2 represents the first peak of NMDA-dependent spike during fast wave propagation. (**g**) The transmembrane potentials of both apical and basal dendrites for three cells (35th Cell, 100th Cell, and 150th Cell along the longitudinal axis).

On the basis of our experimental results, the feasibility that both a fast traveling spike and slow moving waves could co-exist was simulated in Fig. 7a with a model consisting of an array of 200 × 18 pyramidal cells connected only through the ephaptic coupling. Each cell had three compartments: passive soma, apical dendrites capable of generating calcium spikes^20,21^, and basal dendrite capable of generating NMDA spikes since many experiments about NMDA spikes were targeted in the basal dendrites^22,23^. Fast and slow waves can be generated spontaneously in the model and traveled at speeds of approximately 0.1 m/s and of 0.008 m/s respectively similar to those observed experimentally. The fast traveling spike was selectively observed by filtering the raw simulated signals in Fig. 7a with a 1 Hz high-pass filter and retained similar amplitude and speed (Fig. 7b). Similarly, the slow moving wave can be observed by filtering with a 1 Hz low-pass filter and also retained similar magnitudes of the amplitude and speed (Fig. 7c).

To compare the characteristics of simulated and experimental traveling waves, 65 simulated waves were generated by the randomized cell to cell distance *d*_*c-c*_ (2~4 μm) and physiologically relevant stacking factor values (SF = 10~30) based on hippocampal cell density^8^. The firing frequency of the spontaneous spikes was found to be between 0.5 and 1 Hz with a mean value of 0.7 ± 0.3 Hz. The speed of the fast traveling spike was in the range from 0.04 m/s to 0.2 m/s with a mean value of 0.1 ± 0.01 m/s. The slow moving wave origin and direction of propagation was similar to that of the fast one. The propagating speed ranged between 0.004 m/s to 0.02 with a mean value of 0.009 ± 0.0008 m/s. The ratio of propagation speeds between fast and slow waves was limited in the range between 8 to 11 with a mean value of 10 ± 1.1, and the ratios of amplitudes between fast and slow propagations were in the range from 9 to 13 with a mean value of 11 ± 2. The simulated traveling waves were characterized by similar firing frequency and propagating speeds compared to experimental data (Fig. 7d). Simulated extracellular field amplitudes for the fast spikes ranged between 2.74 mV/mm and 5.08 mV/mm (Fig. 7e) and these values fall within the previously observed field amplitude range of ~3~6 mV/mm from 4-AP induced epileptiform activities *in vitro* hippocampal slice^5,6,8,24,25^. It was also determined that the field amplitude increased with stacking factor (SF) and the curve (Field vs SF) shifted to the right when the cell to cell distance (*d*_*cc*_) increased from 2 μm to 4 μm in Fig. 7e. For the larger *d*_*cc*_ (3 and 4 μm), the field amplitudes were lower than 1.0 mV/mm in the region of small values of SF (<21) with no waves generated in the simulated network.

To further study the underlying mechanism of the coexistence of fast and slow moving waves, we analyzed the intracellular membrane potentials in the simulated network. Figure 7f shows the propagation of intracellular potentials in the apical dendrites with calcium channels and in the basal dendrites with NMDA channels. The black bold line indicates the path of NMDA-dependent spike and the green bold line indicates the path of the calcium-dependent spike. Figure 7g shows that the transmembrane potentials of both apical and basal dendrites for three cells (35^th^ Cell, 100^th^ Cell, and 150^th^ Cell along the longitudinal axis). These simulated data clearly show that calcium-dependent spikes generated the slow propagating wave in apical dendrite while NMDA-dependent spikes generated the fast propagating wave in the basal dendrite. Both waves could propagate along the longitudinal axis of the array of the cell. Moreover, the intracellular propagation speeds of calcium-dependent and NMDA-dependent spikes are consistent with the propagating speeds of slow moving wave and fast traveling spikes recorded in the experiments (Fig. 7d). The expanded window of two coexisting intracellular spiking propagations (Fig. 7f) indicates that the origin of NMDA-dependent spike located at P2 (35^th^ Cell) followed the onset of the calcium-dependent spike. The spontaneous calcium-dependent spikes evoked by electric field effect in apical dendrites of several neighboring cells could depolarize slightly membrane potentials in basal dendrites. This small depolarization was further amplified by electric field effects, thereby activating NMDA channels. This activation generated an NMDA-dependent spike and a propagating wave via the ephaptic coupling. Overall, these simulated results strongly support our proposed hypothesis that the calcium-dependent slow moving wave is the source for the NMDA-dependent fast traveling spikes.

## Discussion

By taking advantage of the state-of-the-art voltage-sensitive protein and calcium imaging methods, two different types of traveling waves were detected and traced in longitudinal hippocampal slices. The fast traveling spikes refer to the 4-AP induced inter-ictal epileptiform activity propagating at a speed of approximately 0.1 m/s and also previously observed in the unfolded hippocampus^5^. Most of the fast traveling waves originated in the temporal region of the longitudinal hippocampal slice indicating that the temporal area of the hippocampus is more epileptogenic that the septal side. In fact, several studies have shown that temporal hippocampus is more excitable and more epileptogenic by electrical or chemical stimuli than septal hippocampus^26–28^. In addition, the slow moving waves previously shown to be electrically silent were observed by applying imaging techniques^6^. The presence of this neural source was previously inferred from isochrones lines indicating the arrival of the spikes at the electrodes but could not be observed electrically even when positioned on top of recording sites^6^. The slow wave was revealed by detecting the intracellular calcium concentration in the neurons during propagation of the slow event and the very low frequency bandwidth (<1 Hz) could explain why it was difficult to detect with electrical recording setup. An electrical silence source also documented in cardiac-muscle tissues shows that a spiral wave could propagate through the heart with a speed of 0.1 m/s with the core of the spiral waves electrically silent and move slowly^29^. In the present study, calcium imaging experiments reveal that a neural calcium wave was always accompanied by a fast propagating spike recorded electrically and was with similar speed and direction of propagation already reported in the previous study^6^. Taken together, these data indicate that the slow propagating neuronal calcium wave is the moving source of the spikes. The probability distribution of the delay and speed of the slow moving wave in the presence of APV does not follow a normal distribution (Fig. 5d,e) and this non-Gaussian distribution likely results from the small number of samples. However, a non-parametric statistical analysis does support the hypothesis that the slow moving wave can persist in the presence of NMDA blockers.

Intracellular calcium wave traveling from cell to cell has been shown to be mediated by gap junction or ATP release^17,30^. Our previous study showed that 4-AP induced slow source could still propagate in the presence of gap junctions blockers^5^. The results of this study further show that the slow moving wave continues to propagate in the presence of ATP receptor blocker (Fig. 6c). Therefore, gap junctions and ATP release cannot explain the propagation of the slow moving wave.

Extracellular potassium wave has been found in the adult hippocampus and thus the diffusion of potassium could perhaps explain the propagation of the slow moving wave^31^. However, most activity related to potassium diffusion in the literature demonstrated very slow propagating speeds^31–34^. The speed of the propagation is from 0.00018 to 0.00084 m/s. The speed of potassium diffusion is 10 and even 100 times less than the speed of the slow wave in this study. To the best of our knowledge, the speed of potassium diffusion wave is too slow to explain the slow wave propagation speed.

Based on the experimental results, the propagation of the slow moving wave is different from NMDA-dependent spike, not dependent on the ATP release, and not coupled with the potassium diffusion. However, our modeling simulation suggests that the slow moving wave could propagate through the ephaptic coupling similar to the fast traveling spikes^5^. Calcium signals can generate spikes relying on the calcium-dependent channels, propagate, and generate a wave^35,36^. Therefore, the slow moving wave could be explained by large calcium spikes in many cells generating a self-propagation wave.

The calcium imaging experiments did reveal the propagation trajectory of the slow moving wave based on the analysis of optical signals extracted from different regions of interest. However, the calcium dye (OGB-1) is too slow to image the fast traveling spikes. The causal relationship between the fast traveling spike and slow moving wave cannot be directly established in the calcium imagining experiments. The hypothesis that the fast traveling spikes are generated by the slow moving wave was supported by experiments that combine voltage imaging technique, electrophysiology experiments^6^, and modeling simulation. However, a more definite experiment to establish the causality would require imaging separately each of these two waves.

The model simulation suggests that the slow moving wave is the source of the fast traveling spikes. The computational model including of NMDA channel and calcium channel could generate two separate traveling waves containing similar characteristics compared to the experimental data (Fig. 7d). Moreover, the simulation results show that the fast traveling spikes are mediated by NMDA channels and the slow moving wave is mediated by calcium channels. To further demonstrate the relationship between the fast traveling spikes and the slow moving wave, the model simulation indicates that the NMDA-dependent spike could be triggered by the calcium-dependent spike. However, the NMDA-dependent spike has a faster dynamic than calcium-dependent spike^20,23^ and thus propagates faster and appeared before the calcium-dependent spike reached the peak located at P1 in Fig. 7f. A fully observable calcium spike would misleadingly appear to follow the fully observed NMDA spike (Fig. 7g). Therefore, the fast traveling spikes appear to propagate first and are followed by the calcium wave in the voltage imaging experiments (Fig. 3e) but it should be noted that the fast traveling spikes were triggered by the initiation of the slow moving wave based on the model simulation. The model results, therefore, show that the calcium spike is the source of the NMDA-dependent spikes.

In human patients, it has been found that seizures could spread with slow dynamics^37–40^. The speed of the seizure recruitment varies from 0.004 m/s to 0.01 m/s and the slow moving wave in this study propagated with a similar speed. Therefore, the present *in-vitro* study implies that a slow moving wave might be related to the seizure progression in the early stages. In particular, human seizures can be distinguished by ictal wavefront activity and ictal core activity with the ictal wavefront propagating at a slow speed^39^. Also, a recent study shows that seizures could underlie a spinning wave dynamics^40^. The seizure takes tens of seconds to propagate and the direction of the seizure propagation can be predicted by the slow drift of the signal^4,39^ while the slow neural source in the present study also has very slow dynamics. This phenomenon was also observed in our previous finding that a neural source is moving and generates spikes in different areas^6^. Several studies from patients with epilepsy show that the slow-moving ictal wavefronts can predict the propagation of seizures and the ictal wavefront is related to the inhibitory restraint of seizure activity^4,39,41^. The role of the slow moving wave in this study could be similar to the ictal wavefront since this slow moving wave could trigger the fast traveling spikes and also predict the generation of the fast traveling spikes in other areas^6^. Therefore, the *in-vitro* slow neural source shares many characteristics with the slow waves found in the human brain during seizures.

In summary, we observed two types of the traveling waves in the unfolded hippocampus preparation. One is a fast traveling spike that is related to the inter-ictal activity. The fast traveling spikes could be detected by electrical recording. The other is a slow moving wave that is associated with the neural source of the inter-ictal activity. This slow neural source is neither NMDA nor ATP dependent, and likely not coupled with potassium diffusion. Furthermore, our model simulation suggests that the slow traveling neural source is consistent with calcium spikes propagating by electrical field like epileptiform activity. The slow neural source could possibly explain the slow recruitment of seizures in the early stage or the multi-sites of epilepsy foci.

## Methods

### Animals

VSFP-Butterfly 1.2 transgenic mice were used to perform the experiments of detecting both fast traveling spike and slow moving wave. CD-1 mice were used to perform the experiments of calcium imaging. Mice used for *in-vitro* hippocampal slice studies were approximately 10–30 days old (P10-P30). All experimental procedures performed in this study followed the NIH animal use guidelines and were approved by the Institutional Animal Care and Use Committee (IACUC) at Case Western Reserve University.

### Preparation of longitudinal hippocampal slice

Mice of either sex with ages ranging from P10–P30 were anesthetized by isoflurane and euthanized by decapitation. Next, the brain was removed rapidly from the skull and was cooled rapidly (0–5 °C) in high-sucrose artificial cerebrospinal fluid (S-aCSF) containing (in mM): sucrose, 75; NaCl, 85; KCl, 2.5; NaH2PO4, 1.25; NaHCO3, 25; D-glucose, 25; MgCl2, 4; CaCl2, 0.5; and bubbled with a 95% O2/5% CO2 gas mixture. The hippocampus was separated from the brain by using customized glass pipette tools on a cooling plate. The hippocampus was then sandwiched between two agar gels cut to appropriate size and fixed to a cutting block using cyanoacrylate glue. 400 μm slices were cut longitudinally in S-aCSF at low temperature using a vibratome (VT1000S, Leica, Germany) and placed in a bubbled normal aCSF at room temperature containing (in mM): NaCl, 125, KCl, 2.5; NaH2PO4, 1.25; D-glucose, 25, NaHCO3, 25; MgCl2, 2; CaCl2, 2, to recover for at least 1 h. After one-hour incubation, slices were transferred to a staining chamber for loading the calcium dye or to a bath-immersion recording chamber (Warner Instruments, USA) and superfused with gas-bubbled normal aCSF heated to 32 °C for the further experiments.

### Loading of calcium dye

The acetoxymethyl (AM) ester of the calcium-sensitive dye Oregon Green 488 Bapta-1 (Thermo Fisher Scientific Inc) was used as a calcium indicator. A solution of the dye was prepared in dimethyl sulfoxide (DMSO) to a final concentration of 4 mM. DMSO was used to solubilize the water-insoluble dye. After one-hour recovery of hippocampal slices, a single slice was selected, by visual inspection to identify tissues with prominent cell layers, and transferred to another walled loading chamber containing 3 mL bubbled normal aCSF. The dye solution was loaded directly onto the tissue surface, resulting in a high initial concentration of the dye, but achieving a final concentration of 15 µM^42^. The incubation was performed in dark conditions at room temperature, with the loading time of 10 min plus as many minutes as the age of the mouse in post-natal days^12^. After loading the dye, the slice was placed in the bath-immersion recording chamber and superfused with gas-bubbled normal aCSF. The normal aCSF washed the tissues, resulting in the removal of occurrences of bright spots created by uneven retention of the dye on the surface of the tissue. Imaging and electrical recording began no sooner than 30 min after the tissue was placed in the bath-immersion recording chamber.

### Electrical and optical recording

Glass pipette recording electrodes were pulled to a resistance of 5 MΩ for electrodes used to measure field potentials (150 mMNaCl pipette filling solution). The position of the electrode was manipulated using a micromanipulator (MPC-200, Sutter Instruments), whose range of motion was in all three axes. The glass electrode was positioned on the cell layer, approximately 20 µm below the surface of the tissue, using a 5× objective (Zeiss Microscopy, USA) on the microscope (Zeiss Microscopy, USA) to determine the exact location. The signals were received on an amplifier (Axopatch 200B, Molecular Devices), with amplification at 100. The signals were digitized at 20 kHz sampling frequency by using a digitizer (Digidata 1440 A, Molecular Device), and stored on a computer for analysis.

OGB-1 has an emission wavelength of 494 nm, and an excitation wavelength of 523 nm. To fulfill these criteria, the excitation filter was 488 nm, the emission filter was 520 nm, and the dichroic mirror had a separation wavelength of 516 nm (Semrock, USA). For the optical recording of VSFP-Butterfly 1.2, two different filter sets are prepared for the mCitrine and mKate2. The recording optics included the following filters and splitter: FF01-483/32-25 for mCitrine and mKate2 excitation (Semorck), FF01-542/27 for mCitrine emission (Semorck), BLP01-594R-25 (Semorck) for mKate2 emission, 515LP as the beam splitter for mCitrine, and 580LP as the splitter for mKate2. A broad-spectrum LED light source (X-cite 120LED, Excelitas Technologies) was used during the course of the experiment.

The images were acquired by using a digital CMOS camera (C11440, Hamamatsu Photonics) at a frame rate of 200 Hz (2048 × 512 pixels) for the experiments of calcium imaging and at a higher frame rate of 800 Hz (512 × 64 pixels, 4 × 4 binning) for the experiments of voltage-sensitive imaging. The imaging data were analyzed with MATLAB and signal process toolbox (Math Works).

### Image processing and data analysis

All the acquired image sequences were filtered using a 3 × 3 spatial filter to eliminate electron noise from the camera and shot noise from the acquisition electronics. The background fluorescence is eliminated by subtraction of a background pixel (edge pixel) from each pixel in the entire stack. The fluorescent signals were presented as a percentage of fluorescent change ΔF/F_0_ which was calculated as (F − F_0_)/F_0_, where F_0_ is baseline fluorescence signal averaged over the whole recording period. The image sequence can be considered as pseudo 3-dimensional (3D) signals, with each frame as an image on the x and y-axes, over time, which is the third axis. Hence, each pixel has a single x and y coordinate, and a frame length of time coordinates. The change in the intensity of each pixel value can be mapped over time by inverting the axes such that a single pixel in each frame is shown as the intensity changes in the fluorescence over time.

To determine the speed of propagation of the traveling waves, a cross-correlation measurement was used to estimate the delay between two optical signals from two regions of interest. The regions were selected manually to restrict selection only on the visible cell layer. All measurements were taken from the temporal to the septal side of the tissue. If the delay was positive, the direction of propagation was defined as temporal to septal.

For mapping, the dynamics of calcium in the glial cell, the regions of interest in glial cells were identified manually based on the criteria that the fluorescent intensity is twice higher than the baseline intensity. The optical signal was reconstructed in the time domain to trace the delay between two glial cells. The wavefront, instead of the peak, of the signal, was used to measure the speed of propagation between signals. A cross-correlation measurement was used to quantify the delay between signals.

### Statistical analysis

A statistical analysis was done by using t-test to compare the speeds in two different conditions. A statistical significance criterion of α = 0.05 was used for all tests. Results are shown as mean ± the standard deviation of the mean. The calculation was based on events unless noted.

### Computational modeling

#### Single cell model

The three-compartmental model (Fig. S2a) for a hippocampal pyramidal cell (HPC) was built under Matlab stimulation environment. This model was tested for 4-AP condition-induced both NMDA-dependent fast epileptiform propagation and Calcium-dependent slow wave propagation. In the model, each cell contains three compartments, with one compartment for soma, one compartment for the apical dendrite and one compartment for the basal dendrite. Similar as to previous models, the soma has an area of 995.4 μm^2^ with a diameter of 17.8 μm, and, the other two dendritic compartments have the same diameter of 5.2 μm and length of 735.3 μm for the apical one, and 490.2 μm for the basal one^43,44^. In our model, we set about half lengths of dendrites i.e. 400 μm for the distance between apical dendritic and somatic compartment, and 250 μm for the distance between basal dendritic and somatic compartment, respectively. The passive HPC membrane parameters were set to the following values: membrane resistance *R*_*m*_ = 680 *Ω*·*cm*^2^ and membrane capacitance *C*_*m*_ = 1.0 *μF*/*cm*^2^ for both somatic and dendritic compartments, and axial resistance *R*_*i*_ = 530 *Ω*·*cm*. With these parameters, the electronic parameters for each compartment can be determined by cable theory, as listed in Table S1.

It is well known that NMDA receptors are widely distributed in branches of dendrites e.g. basal, oblique and tuft termed as “non-apical” dendrites while calcium channels dominantly locate at the apical trunk of dendrite^22^. To simplify the following network model, NMDAR channels and Ca channels were separately included in basal and apical dendrite in the current proposed model (Fig. S2a). NMDA receptors were not considered in apical dendrite because NMDA receptors located in distal apical dendrite far away from the soma, compared with those in the basal dendrite, generated weaker field effects on the neighbor neurons. Moreover, basal dendritic compartment contained a delayed rectifier potassium current *I*_*KDR*_ and apical dendritic compartment contained a short-duration Ca- and voltage-dependent potassium current *I*_*KC*_ and a long-duration Ca-dependent current potassium current *I*_*KAHP*_. In contrast with the previous models, the somatic compartment was only considered as a passive one. It is worth noting that Na channels were not considered at soma in current model for the reason that this model was purposed to verify whether and how those coexisting fast and slow propagation behaviors observed *in vitro* experiments are evoked by interactive dendritic activities mediated via NMDA and Ca channels.

The transmembrane potentials (*V*_*m*_*ad*_, *V*_*m*_*s*_, and *V*_*m*_*bd*_) for three compartments of the model is described by the relationship

1 $${C}_{m}\frac{d{V}_{m\text{\_}ad}}{dt}=-{I}_{Ca}-{I}_{KC}-{I}_{KAHP}-{I}_{L\text{\_}ad}-\frac{{{g}}_{c\text{\_}s\text{\_}ad}}{{A}_{ad}}({V}_{m\text{\_}ad}-{V}_{m\text{\_}s})$$

2 $${C}_{m}\frac{d{V}_{m\text{\_}s}}{dt}=-{I}_{L\text{\_}s}-\frac{{g}_{c\text{\_}s\text{\_}ad}}{{A}_{s}}({V}_{m\text{\_}s}-{V}_{m\text{\_}ad})-\frac{{g}_{c\text{\_}s\text{\_}bd}}{{A}_{s}}({V}_{m\text{\_}s}-{V}_{m\text{\_}bd})$$

3 $${C}_{m}\frac{d{V}_{m\text{\_}bd}}{dt}=-{I}_{NMDA}-{I}_{KDR}-{I}_{L\text{\_}bd}-\frac{{g}_{c\text{\_}s\text{\_}bd}}{{A}_{bd}}({V}_{m\text{\_}bd}-{V}_{m\text{\_}s})$$

The gating equations for each active current are implemented using the Hodgkin-Huxley formalism as in Table S2. The channel conductance is *g*_Ca_ = 20 ms/*cm*^2^, ${\bar{g}}_{NMDA}=5.6\,{\rm{mS}}/c{m}^{2}$, *g*_*KC*_ = 5 mS/*cm*^2^, *g*_*KAHP*_ = 0.3 mS/*cm*^2^, and *g*_*KDR*_ = 200 mS/*cm*^2^. The reversal potentials are *E*_*Ca*_ = 70 *mV*, *E*_*NMDA*_ = 0 *mV*, *E*_*K*_ = −60 *mV*, *E*_*L_d*_ = −58 *mV*, and *E*_*L_S*_ = −70 *mV*.

The kinetics of gating variables *x* for *I*_*Ca*_ and *I*_*KC*_ in apical dendrite are governed by the following equation:

4 $$\frac{dx}{dt}=\frac{{x}_{\infty }({V}_{m\text{\_}ad})-x}{{\tau }_{x}}$$

with (i.e. *x* ∈{*m*_*Ca*_, *h*_*Ca*_, *c*}. The activation and inactivation steady-state function ***x***_∞_ are given in Boltzmann form.

5 $${x}_{\infty }=\frac{1}{1+\exp (-({V}_{m\text{\_}ad}-{\theta }_{x})/{k}_{x})}$$

The half activation/inactivation voltages *θ*_*x*_, slope rates *k*_*x*_ at *θ*_*x*_ and time scales *τ*_*x*_ are all constant shown in Table S3.

The dynamics of the calcium concentration inside the HPC, [*Ca*^2+^]_*i*_ is described as

6 $$\frac{d{[C{a}^{2+}]}_{i}}{dt}=-v{I}_{Ca}-\frac{{[C{a}^{2+}]}_{i}}{{\tau }_{[Ca]}}$$

with the constant values of *v* and *τ*_*Ca*_ shown in Table S3. The variable [*Ca*^2+^]_*i*_ in our model is dimensionless^45^. It is proportional to the *Ca*^2+^ concentration in a thin internal cylindrical shell adjacent to the membrane. [*Ca*^2+^]_*i*_-dependent gate variable χ(*Ca*) is given by a literature^46^.

7 $${\rm{\chi }}(Ca)=\frac{1}{1+{a}_{c}/{[C{a}^{2+}]}_{i}}$$

where the constant value of *a*_c_ is shown in Table S3.

Gate variable *q* depends on its corresponding gate function

8 $$\frac{dq}{dt}=\frac{{q}_{\infty }({[C{a}^{2+}]}_{i})-q}{{\tau }_{q}}$$

and steady function *q*_∞_ is the following

9 $${q}_{\infty }({[C{a}^{2+}]}_{i})=\frac{1}{1+{a}_{q}^{4}/{[C{a}^{2+}]}_{i}^{4}}$$

with the constant values of *τ*_*q*_ and *a*_*q*_ shown in Table S3.

The dynamics of gate variable *q* for *I*_*KDR*_ in basal dendrite is described by

10 $$\frac{dn}{dt}={\alpha }_{n}({V}_{m\text{\_}ad})-n\cdot ({\alpha }_{n}({V}_{m\text{\_}ad})+{\beta }_{n}({V}_{m\text{\_}ad}))$$

with the variable rate functions modified from the literature^47^

11 $${\alpha }_{n}=\frac{0.00049\cdot ({V}_{m\text{\_}ad}-32)}{1.0-exp(-\frac{{V}_{m\text{\_}ad}-32}{25.0})},\,{\beta }_{n}=\frac{0.00008\cdot ({V}_{m\text{\_}ad}-42.0)}{exp(\frac{{V}_{m\text{\_}ad}-42.0}{10.0})-1.0}$$

In the case of the NMDA-gated channel, there is a marked voltage-dependency in the presence of extracellular magnesium. For physiological magnesium concentrations, the dependence on voltage of NMDA receptor-mediated current *I*_*NMDA*_ can be integrated in a gating function *B*(*V*_*mbd*_) that multiplies the NMDA conductance ${\bar{g}}_{NMDA}$ (Table S2). This gating function is

12 $$B({V}_{m\text{\_}bd})=\frac{1}{1+\frac{{[M{g}^{2+}]}_{o}}{{M}_{0}}\exp (-{k}_{B}\cdot {V}_{m\text{\_}bd})}$$

where [*Mg*^2+^]_*o*_ is the extracellular *Mg*^2+^ concentration of 1 mM and *M*_0_ and *k*_*B*_ are constants shown in Table S3. It should be noted that ${\bar{g}}_{NMDA}$ in this model is proportional to fraction of NMDA channels in the open state *O*_*NMDA*_ i.e. ${\bar{g}}_{NMDA}={g}_{NMDA}\cdot {O}_{NMDA}$, where *g*_*NMDA*_ is the maximum conductance of NMDA channel. Though the kinetics of *O*_*NMDA*_ is complex, it’s a nearly constant when the synaptic events in HPC dendrite are considered as the independently-generated random Poisson processes with the same frequency. Thus, to focus on the fast/slow propagation mediated by extracellular electric field, it is reasonable that ${\bar{g}}_{NMDA}$ is considered as a constant under some stable synaptic activities background. The values of all parameters for single HPC are taken from above values unless otherwise specified.

#### HPC neural network

The layout of the network was designed in a way to capture the most features of both fast and slow propagation activities in physiological cellular layout. We simulate the hippocampal pyramidal cell (HPC) network in physiological CA1 region with 4000 μm(X) × 600 μm(Y) × 360 μm(Z) (Fig. S1a), where X and Z represent longitudinal and transverse directions of hippocampus respectively, and Y represents the tissue thickness along the cellular axial direction. One stack network of this region is made up of cell arrays with 200 neurons in X-axis and 18 neurons in Z axis (Fig. S1b,c). The distance between every two adjacent neurons (*d*_c-c_) in a physiologic pyramidal network ranges from 2 to 5 μm^48^. In this paper, we tested *d*_c-c_ from 2 to 4 μm and its values were assigned in the text. To represent the cell stacking throughout the depth of a tissue slice, similar to the handling method in Chen Q *et al*., 2015^8^, a “stacking factor” (SF) was used to take into account the actual number of cells around one stack network (Fig. S1d). Here, the value of SF is in the range between 10 and 30^8^.

#### Electric field coupling

To test the hypothesis that endogenous electric field alone could induce both fast and slow neural physiologic propagation observed *in vitro*, the synaptic connections, gap junction, and diffusion effects were eliminated in the network while communications between adjacent cells were implemented by electric field coupling. To obtain the higher computational efficiency, electric field effect could only propagate action potentials in the longitude direction (X-axis) i.e. the cells in any two different columns (Z axis) were coupled bidirectionally while those in each transverse direction (Z axis) were not affected each other. The electric field effect was calculated using the quasistatic formulation of the Maxwell equations assuming homogeneous and linear volume conductors. According to Ohm’ law, the corresponding potential *φ* at the point P at distance *r* relative to reference electrode in a medium of resistivity *ρ* is described as follow:

13 $$\phi =\frac{\rho }{4\pi r}\cdot I$$

Using superposition, Eq. (13) can be generalized to monopolar electrodes from each of three compartments from a source Cell_(*i,j*)_ in the network array with transmembrane currents *I*_*i,j*___*tran_ad*_, *I*_*i,j*___*tran_s*_, and *I*_*i,j*___*tran_bd*_ at the corresponding distances to each of three compartments of the target Cell_(*k, l*)_ (j≠*l*) in the cell array. The extracellular potential inserted at three compartments of the target Cell_(*k, l*)_ is given by the following:

$${V}_{(k,l)\text{\_}e\text{\_}z}=SF\cdot \frac{\rho }{4\pi }\sum _{(i,j)}(\frac{{I}_{(i,j)\text{\_}tran\text{\_}ad}}{{r}_{(i,j)\text{\_}ad\to k,l\text{\_}z}}+\frac{{I}_{(i,j)\text{\_}tran\text{\_}s}}{{r}_{(i,j)\text{\_}s\to (k,l)\text{\_}z}}+\frac{{I}_{(i,j)\text{\_}tran\text{\_}bd}}{{r}_{(i,j)\text{\_}bd\to (k,l)\text{\_}z}})\quad (z=ad,s,bd)$$

where *V*_(*k,l*)_*e*_*z*_ is the extracellular potential inserted at target compartment *z* in the target Cell_(*k, l*)_; *I*_(*I,j*_)*_*_*tran_z*_ is the transmembrane current of three compartments (assuming current going out of the cell to be positive direction) in source Cell_(*i,j*)_ located *r*_(*i,j*)_*ad*→*k,l_z*_, *r*_(*i,j*)_*s*→(*k,l*)*_z*_ and *r*_(*i,j*)_*bd*→(*k,l*)*_z*_ distances from the target compartment z (Fig. S2); *ρ* is the extracellular resistivity with the range of 250~380 *Ω*·*cm* and here 300 *Ω*·*cm* was used in the model^20,49–51^ and SF is the stacking factor.

#### Extracellular potential, Field amplitude, and speed measurement

To measure the resulting extracellular potential and electric field due to network spiking activities and compare them to the experimentally recorded waveforms *in vitro*, we placed 2 × 3 virtual multi-electrode array outside of the network paralleling to (X, Y) plane (Fig. S3), similar as to the placement of the measuring electrodes into the *in vitro* slice. The virtual electrode array was placed about 30 *μm* away from the surface layer of the rectangular cell array to account for approximately three rows of the dead cell around inserted electrodes, a situation observed experimentally. All cells’ resulting extracellular potential at the multi-electrode array are calculated, and field amplitude was calculated by finding the spatial derivatives of extracellular potentials along the Y-axis. The network field amplitude was the average of the field amplitudes at three different positions (Eq. (15), i.e. Temporal, Middle and Septal, Fig. S3)

15 $${E}_{network}=\frac{\frac{{V}_{e\text{\_}Tb}-{V}_{e\text{\_}Tt}}{2}+\frac{{V}_{e\text{\_}Mb}-{V}_{e\text{\_}Mt}}{2}+\frac{{V}_{e\text{\_}Sb}-{V}_{e\text{\_}St}}{2}}{3}$$

The propagation speeds including fast and slow ones measured based on the three extracellular recording at the top of virtual electrode array i.e. *V*_*e_Tt*_, *V*_*e_Mt*_ and *V*_*e_St*_, where peak times for both fast and slow propagation oscillations are recorded, and the delay from *T*_*t*_ to *M*_*t*_ and from *M*_*t*_ to *S*_*t*_ were calculated. The propagation speed was derived by taking the travelling distance divided by delay time. To determine the coexistence of the fast and slow propagations, the filtered virtual extracellular recordings were obtained via 1 Hz high-pass Butterworth filter and 1 Hz low-pass Butterworth filter respectively similar as to those used in the experiments.

### Data availability

The datasets generated during and/or analyzed during the current study are available from the corresponding author on reasonable request.

## Electronic supplementary material

Supplementary Information
