# Supplementary material for: Slow moving neural source in the epileptic hippocampus can mimic progression of human seizures
Source: Sci Rep. 2018 Jan 24;8:1564. doi: 10.1038/s41598-018-19925-7 (PMC5784157; doi:10.1038/s41598-018-19925-7)
Supplement: Supplementary file 1 — Supplementary Information [file 41598_2018_19925_MOESM1_ESM.pdf]

Slow moving neural source in the epileptic hippocampus can mimic progression of human seizures

Chia-Chu Chiang[1], Xile Wei[1,2], Arvind Keshav Ananthakrishnan[1], Rajat S. Shivacharan, Luis E. Gonzalez-Reyes, Mingming Zhang, and Dominique M. Durand*

Affiliation

Department of Biomedical Engineering, Case Western Reserve University, Cleveland, Ohio, 44106 USA.

1. These authors have equal contribution.

2. Currently in the School of Electrical and Information Engineering, Tianjin University, Tianjin, 300072, China.

Supplementary Table 1. The electronic parameters in the computational model.

Parameter	symbol	Value
Area of soma	A_s	$1.0 \times 10^{-5}\ cm^2$
Area of apical dendrite	A_{ad}	$1.2 \times 10^{-4}\ cm^2$
Area of basal dendrite	A_{bd}	$8.0 \times 10^{-5}\ cm^2$
Membrane capacitance	C_m	$1.0\ \mu F/cm^2$
Conductance between soma and apical dendrite	$g_{c\_s\_ad}$	$1.0 \times 10^{-5}\ ms$
Conductance between soma and basal dendrite	$g_{c\_s\_bd}$	$1.6 \times 10^{-5}\ ms$
Membrane leakage conductance for soma and dendrite	g_{m_Leak}	$1.47\ ms/cm^2$

Supplementary Table 2. The Hodgkin-Huxley equations of different channels in the model.

Channel	Gating functions
I_{Ca}	$g_{Ca} \cdot m_{Ca}^2 \cdot h_{Ca}(V_{m\_ad} - E_{Ca})$
I_{NMDA}	$\bar{g}_{NMDA} \cdot B(V_{m\_bd}) \cdot (V_{m\_bd} - E_{NMDA})$
I_{KC}	$g_{KC} \cdot c \cdot \chi(Ca) \cdot (V_{m\_ad} - E_K)$
I_{KAHP}	$g_{KAHP} \cdot q \cdot (V_{m\_ad} - E_K)$
I_{KDR}	$g_{KDR} \cdot n^4 \cdot (V_{m\_bd} - E_K)$
I_{L_ad}	$g_{m\_Leak} \cdot (V_{m\_ad} - E_{L\_d})$
I_{L_s}	$g_{m\_Leak} \cdot (V_{m\_s} - E_{L\_s})$
I_{L_bd}	$g_{m\_Leak} \cdot (V_{m\_bd} - E_{L\_d})$

Supplementary Table 3. Parameter values of HPC model. References and comments: 1) modified from Jakub N et al., 2003[50]. 2) modified from Golomb D. et al., 2006[46]. 3) modified from Destexhe A. et al., 1994[51].

parameter variables	value	References /Comment	parameter variables	value	References/Comment
$\theta_{m_{Ca}}$	-20.0mV	1	τ_c	20	2
$k_{m_{Ca}}$	16.0mV	1	v	$0.13cm^2/(ms \cdot \mu A)$	2
$\tau_{m_{Ca}}$	35ms	1	$\tau_{[Ca]}$	13ms	2
$\theta_{h_{Ca}}$	-40.0mV	1	a_c	6	2
$k_{h_{Ca}}$	-7.0mV	1	a_q	3	2
$\tau_{h_{Ca}}$	157ms	1	τ_q	300ms	2
θ_c	-37.0mV	2	M_0	3.57mM	3
k_c	7.2mV	2	k_B	0.0744 mV^{-1}	3

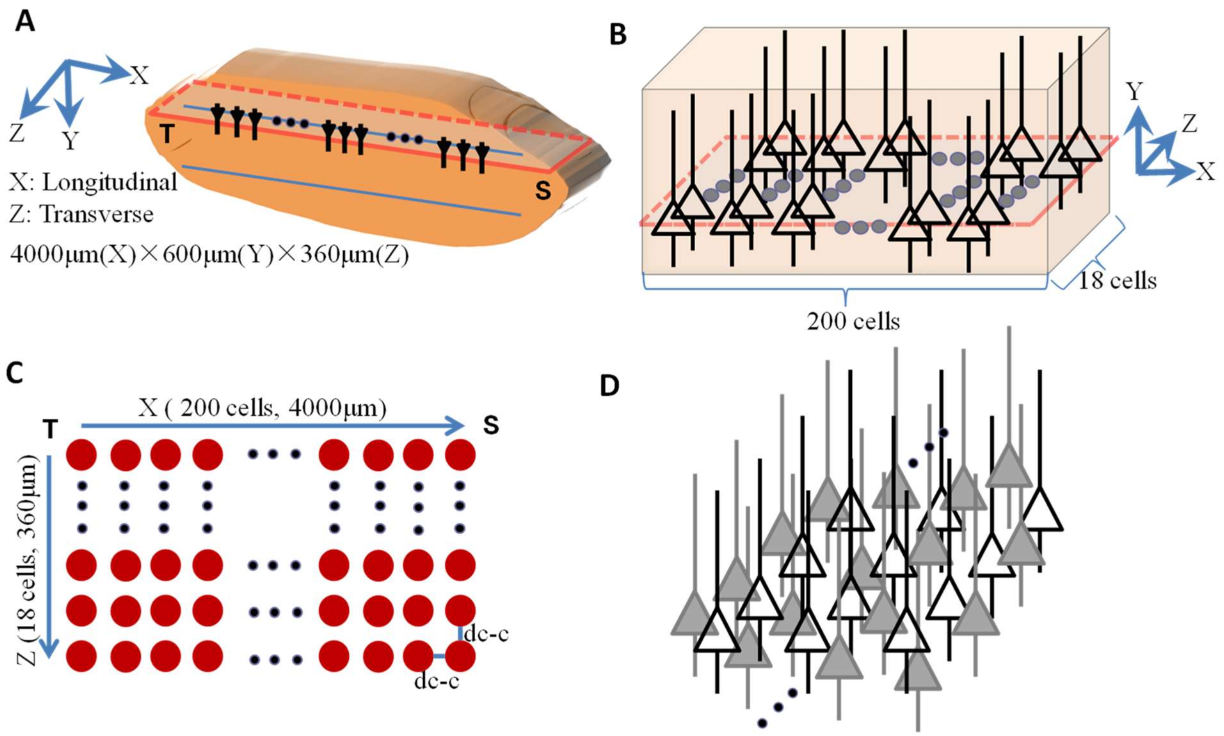

Figure S1. Layout of simulated hippocampal pyramidal cell (HPC) network. (A) 3-D view of the hippocampus slice where simulated region is approximately limited by 4000μm (X)×600 μm(Y))×360 μm(Z). The red rectangle represent one somatic plane layer L_soma of CA1 pyramidal cells. (B) 3-D view of one stack comprised of L_soma contains 200×18 cells in (X, Y) plane. (C) Top view in (X, Y) plane of one stack network (solid-colored circles represent soma position). The diameter of the soma is 17.8μm and the soma edge-to-edge distance dcc ranges from 2 to 4 μm. Thus, the dimension of the network is 3958~4356 μm (X) ×354~388 μm (Y). (D) Physical representation of the stacking factor (SF). White-colored cells represent the actual modeled cell location, whereas gray-colored cells represent the virtually stacked cells around the modeled cell locations. As an example with SF=3, each modeled cell was surrounded by two extra virtual cells at the same location and the amplitude of electric field generated by modeled cells was multiplied by three.

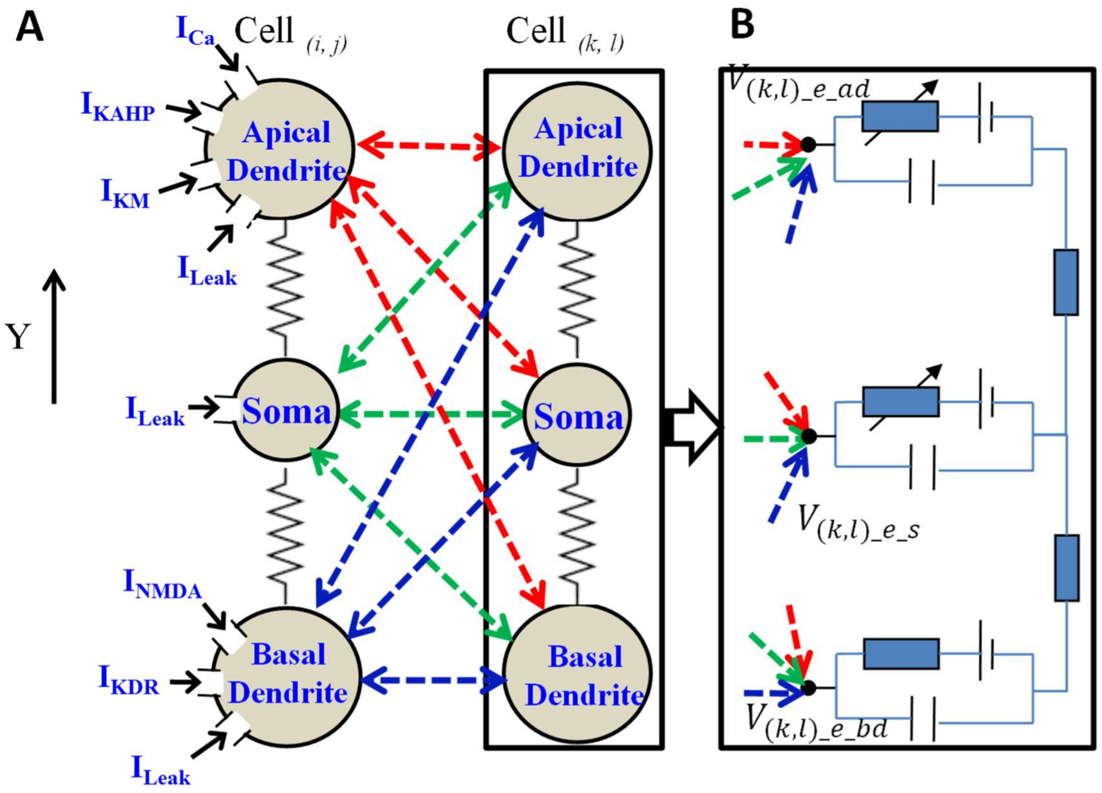

Figure S2. Three-compartmental neuron model and electric field interactions between two cells within the neural network. (A) each modeled cell contained three compartment: apical dendrite (ad), soma (s) and basal dendrite (bd). Three ion channel currents (INMDA, IKDR, ILeak) are added into apical dendritic compartment. Four ion channel currents (ICa, IKAHP, IKM, and ILeak) are inserted into basal dendritic compartment. Somatic compartment is consider as a passive one. Cell(i,j) and Cell(k, l) represented any two cells of network in different column (Z axis) in (X, Z) plane in Figure S1C, where 1≤i,k≤200, 1≤j,l≤18, and j≠l. Electric field couplings between any two compartments among Cell(i,j) and Cell(k, l) were bidirectional. Each paired couplings were symbolized as (i,j)_z→(k,l)_z and (k,l)_z→(i,j)_z where z={ad,s,bd}. (B) Extracellular potentials (V_((k,l)_e_ad), V_((k,l)_e_s), and V_((k,l)_e_bd)) acted on each extracellular node of three compartments of the target Cell(k, l) using Eq. (2). Field effect on each compartment of the target Cell(k, l) was the superposition of all fields generated by all source Cell(i,j).

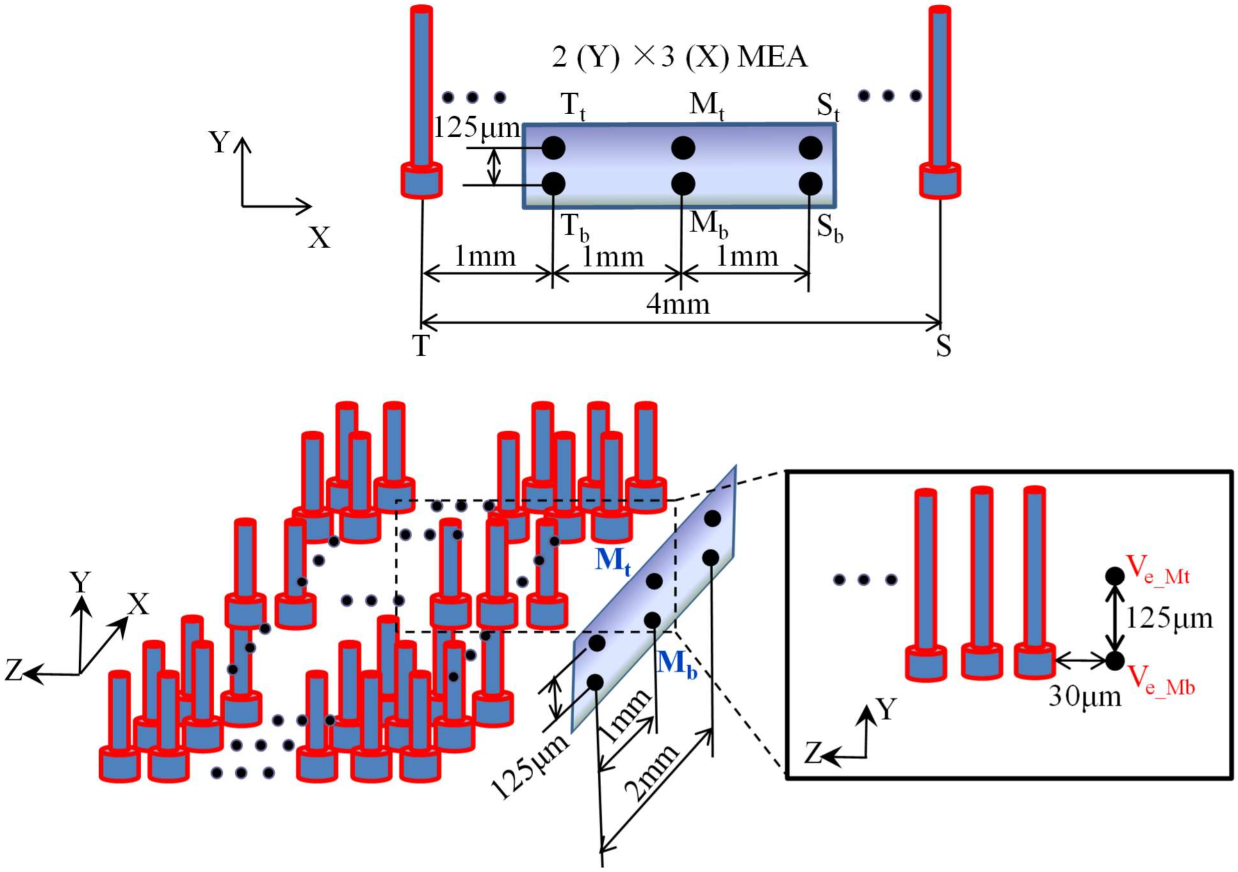

Figure S3. Extracellular potential, field measurements in modeled network. a) A virtual 2×3 multi-electrode array (MEA) is mimicked to match the recording electrodes of in vitro experimental settings. This MEA is placed parallel to and 30μm away from surface layer in (X, Y) plane of the network. The solid black circles are virtual recording electrodes with top ones (Tt, Mt, and St) and bottom ones (Tb, Mb, Sb). The bottom electrodes are placed in the same plane (X, Z) of the somatic layer. The distance between top and bottom electrodes is set as 125μm. Mt and Mb are located in the middle of limited longitudinal scope with 2mm distance from two borders in X axis. (Tt, Tb) and (St, Sb) are located in two sides of (Mt, Mb) with the equal distance of 1mm. Extracellular potential at the site of each electrode was calculated by superposition of fields at this site generated by all cells in network using Eq. (1). Field amplitudes are calculated using Eq. (3).
